# Longitudinal analysis of T cell receptor repertoires reveals shared patterns of antigen-specific response to SARS-CoV-2 infection

**DOI:** 10.1172/jci.insight.151849

**Published:** 2022-05-23

**Authors:** Rachel M. Gittelman, Enrico Lavezzo, Thomas M. Snyder, H. Jabran Zahid, Cara L. Carty, Rebecca Elyanow, Sudeb Dalai, Ilan Kirsch, Lance Baldo, Laura Manuto, Elisa Franchin, Claudia Del Vecchio, Monia Pacenti, Caterina Boldrin, Margherita Cattai, Francesca Saluzzo, Andrea Padoan, Mario Plebani, Fabio Simeoni, Jessica Bordini, Nicola I. Lorè, Dejan Lazarević, Daniela M. Cirillo, Paolo Ghia, Stefano Toppo, Jonathan M. Carlson, Harlan S. Robins, Andrea Crisanti, Giovanni Tonon

**Affiliations:** 1 Adaptive Biotechnologies, Seattle, Washington, USA.; 2 Department of Molecular Medicine, University of Padova, Padua, Italy.; 3 Microsoft Research, Redmond, Washington, USA.; 4 Division of Infectious Diseases and Geographic Medicine, Stanford University School of Medicine, Stanford, California, USA.; 5 Azienda Ospedale Padova, Microbiology and Virology Unit, Padua, Italy.; 6 Division of Immunology, Transplantation and Infectious Disease, IRCCS Ospedale San Raffaele, Milan, Italy.; 7 Department of Medicine, University of Padova, Padua, Italy.; 8 Center for Omics Sciences and; 9 Division of Experimental Oncology, IRCCS Ospedale San Raffaele, Milan, Italy.; 10 Vita-Salute San Raffaele University, Milan, Italy.; 11 CRIBI Biotech Center, University of Padova, Padua, Italy.; 12 Department of Life Sciences, Imperial College London, London, United Kingdom.

**Keywords:** COVID-19, Immunology, T cells

## Abstract

T cells play a prominent role in orchestrating the immune response to viral diseases, but their role in the clinical presentation and subsequent immunity to SARS-CoV-2 infection remains poorly understood. As part of a population-based survey of the municipality of Vo’, Italy, conducted after the initial SARS-CoV-2 outbreak, we sampled the T cell receptor (TCR) repertoires of the population 2 months after the initial PCR survey and followed up positive cases 9 and 15 months later. At 2 months, we found that 97.0% (98 of 101) of cases had elevated levels of TCRs associated with SARS-CoV-2. T cell frequency (depth) was increased in individuals with more severe disease. Both depth and diversity (breadth) of the TCR repertoire were positively associated with neutralizing antibody titers, driven mostly by CD4^+^ T cells directed against spike protein. At the later time points, detection of these TCRs remained high, with 90.7% (78 of 96) and 86.2% (25 of 29) of individuals having detectable signal at 9 and 15 months, respectively. Forty-three individuals were vaccinated by month 15 and showed a significant increase in TCRs directed against spike protein. Taken together, these results demonstrate the central role of T cells in mounting an immune defense against SARS-CoV-2 that persists out to 15 months.

## Graphical abstract

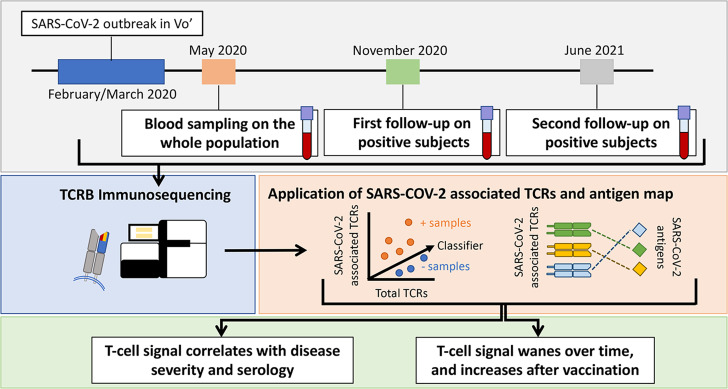

## Introduction

The clinical presentation of and subsequent immunity to COVID-19 are diverse, with a wide range of disease severity and susceptibility to future infection. With vaccines widely available, predicting who is still susceptible to severe disease is now a key public health goal across the globe. Antibody response has been shown to correlate with disease severity (1–4) and with protection from future infection (5–9), but it provides incomplete predictive power, making it important to assess additional factors that could influence these outcomes (10).

The T cell response plays a key role in the clearance of viral infections, governing and orchestrating both cellular and humoral immunity (11). Recent evidence demonstrates that the T and B cell responses can be discordant, and, in some individuals, there is a T cell response without antibody production (12–14). It remains uncertain whether, and for how long, prior infection with SARS-CoV-2 provides immunity against future reinfection, nor is it known how the severity of disease might influence long-term immunity (15). In the related SARS-CoV-1 and Middle East respiratory syndrome (MERS) coronaviruses, infections elicit an enduring T cell response with a more fleeting antibody response (16). Several studies have also shown that the T cell response is diverse, targeting regions across the viral genome, while the B cell response is concentrated against spike protein (17–22). These findings likely underlie recent observations that protection against the virus induced by vaccination remains high against recent emerging variants such as Delta and Omicron, despite large reductions in neutralizing antibody (NAb) titers against these variants (23–26). Finally, genetic factors influencing antigen presentation to T cells have also been shown to impact disease severity (27). These data further reinforce the central role of the T cell response in SARS-CoV-2 infection (28–30). Therefore, direct, quantitative measures of the T cell response to SARS-CoV-2 infection, particularly in longitudinal samples following recovery, may offer crucial insights into immunity and more broadly of the mechanisms underlying the immune response to this virus (11, 17, 31).

While various methodologies for measuring the T cell response exist, immunosequencing is a robust and precise tool that could be deployed at population scale (32–34). Following the first reported COVID-19 death in Vo’, Italy, and subsequent lockdown of the entire municipality, a large PCR-based study was undertaken to screen the majority of the residents in that area in an unbiased manner. Approximately 60 days after the initial PCR survey, blood samples were collected from the majority of these study participants, and quantitative assessment of both SARS-CoV-2–specific T cells and IgG antibody titers were performed. After this unbiased screen of the municipality, additional longitudinal blood samples were collected from positive individuals at 9 months and 15 months in order to characterize persistence of the adaptive immune response over time. This cohort was also finely characterized in terms of demography, clinical presentation, hospitalization, comorbidities, therapies, and contact network (35). This carefully curated data set provides an invaluable opportunity to characterize the role of T cells over time in infection and vaccination.

## Results

### Characterizing the T cell response to SARS-CoV-2.

T cell receptor (TCR) sequencing was performed on blood samples collected 2 months after infection from 2291 residents of Vo’ using ImmunoSEQ. Of those, 76 had a PCR-confirmed diagnosis of COVID-19, and the rest were PCR^–^ at both surveys. Serology data were also available from 3 commercial tests, with 2156 samples having results from all 3 assays, as well as NAb titers for 153 samples. An additional 25 samples were determined to be confidently COVID-19^+^, even if they had negative or missing real-time PCR tests, if they were positive to at least 2 SARS-CoV-2 antigens (nucleocapsid protein and spike protein) based on the serology tests or if they had detectable neutralizing antibodies consistent with the baseline ground truth definition in Dorigatti et al. (36). Another set of 2022 samples that were negative by PCR and all 3 serology tests was considered to be confidently COVID-19^–^ for comparison. Additional contact tracing and symptom data suggest that the 25 samples came from true cases, compared with lower rates of symptoms and household exposure in the confident negative set (Supplemental Figure 1A; supplemental material available online with this article; https://doi.org/10.1172/jci.insight.151849DS1). Of the 10 individuals with symptoms but negative PCR results, 5 were symptomatic prior to the PCR survey, while 5 were symptomatic during the PCR survey and may have been false negatives. In total, 33 of these 101 COVID-19 cases were asymptomatic, 58 had symptoms but did not require hospitalization, and 10 were hospitalized. The majority of COVID-19cases were in patients 50 years of age or older (62%).

**Figure 1:**
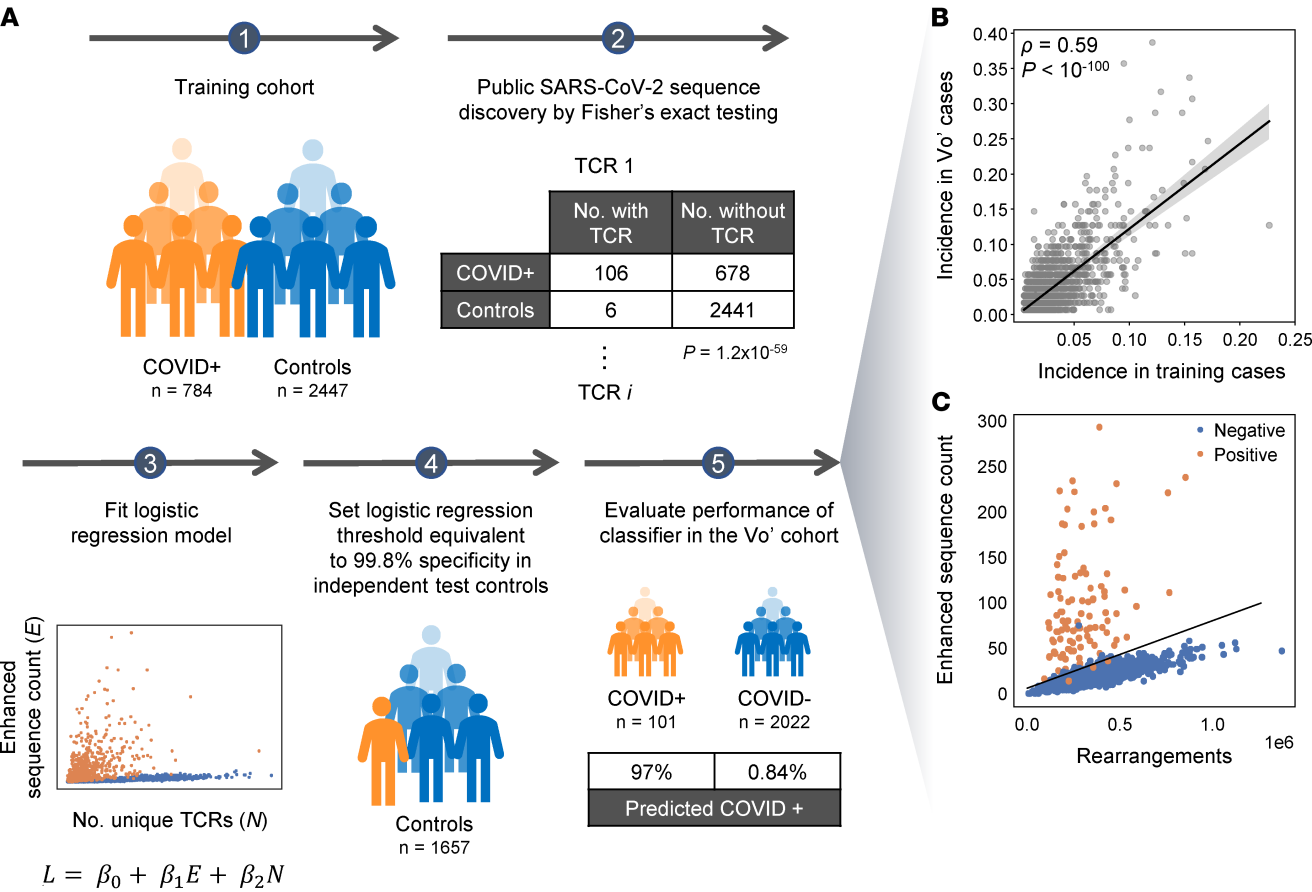
Identification and use of public T cell receptors to characterize the SARS-CoV-2 immune response. (**A**) Schema of the previously generated classification framework, also described in refs. 18 and 33, starting from a case-control design and Fisher’s exact testing for each TCR on independent training data, to identify public TCR sequences that are overrepresented in cases versus controls. Following logistic regression to establish the T cell test threshold for determining recent or past infection, the receptors are applied to this Vo’ study data set. COVID^+^ samples include all 101 samples defined by the positive ground truth samples set from Dorigatti et al. (36), while controls included the 2022 samples that were negative by PCR and all 3 serology tests. (**B**) Incidence of each TCR sequence compared in the training data and in the Vo’ PCR^+^ cases. (**C**) The count of enhanced sequences is plotted versus the total number of unique TCR rearrangements for individuals in the Vo’ study data set that were positive (orange) or negative (blue).

We previously identified 4287 public TCR sequences associated with SARS-CoV-2 using a case/control design that included several cohorts from the United States and Europe (18, 33) and validated their diagnostic accuracy in an independent US-based cohort (Figure 1A). Notably, 58% of these sequences were also present in PCR^+^ Vo’ cases, and 8.2% were found at an incidence of 5% or higher. Overall, the incidence of these sequences was highly correlated across cohorts, indicating that a substantial subset of public SARS-CoV-2–specific TCRs is common across distinct populations (Figure 1B). Using these public sequences and previously defined classification framework (18, 33), 98 of 101 (97.0%) of the COVID-19^+^ individuals had a positive T cell test result, and 74 of 76 (97.4%) of PCR^+^ individuals were positive (Supplemental Table 1). There was substantial variation in the number of public sequences across individuals (Figure 1C), and most of the additional 25 samples without PCR support showed equally high signal (Supplemental Figure 1B). Interestingly, 1 sample from an individual who was symptomatic but negative by all 3 serology assays and PCR had 75 SARS-CoV-2–associated sequences, making it strongly positive by the T cell test. The sample was not evaluated by the neutralization assay but could represent a case in which the T cell response is detectable, even in the absence of a B cell response. Overall, these data indicate that detectable TCR signatures are present 2 months after infection across a range of COVID-19 disease severities.

**Figure 2:**
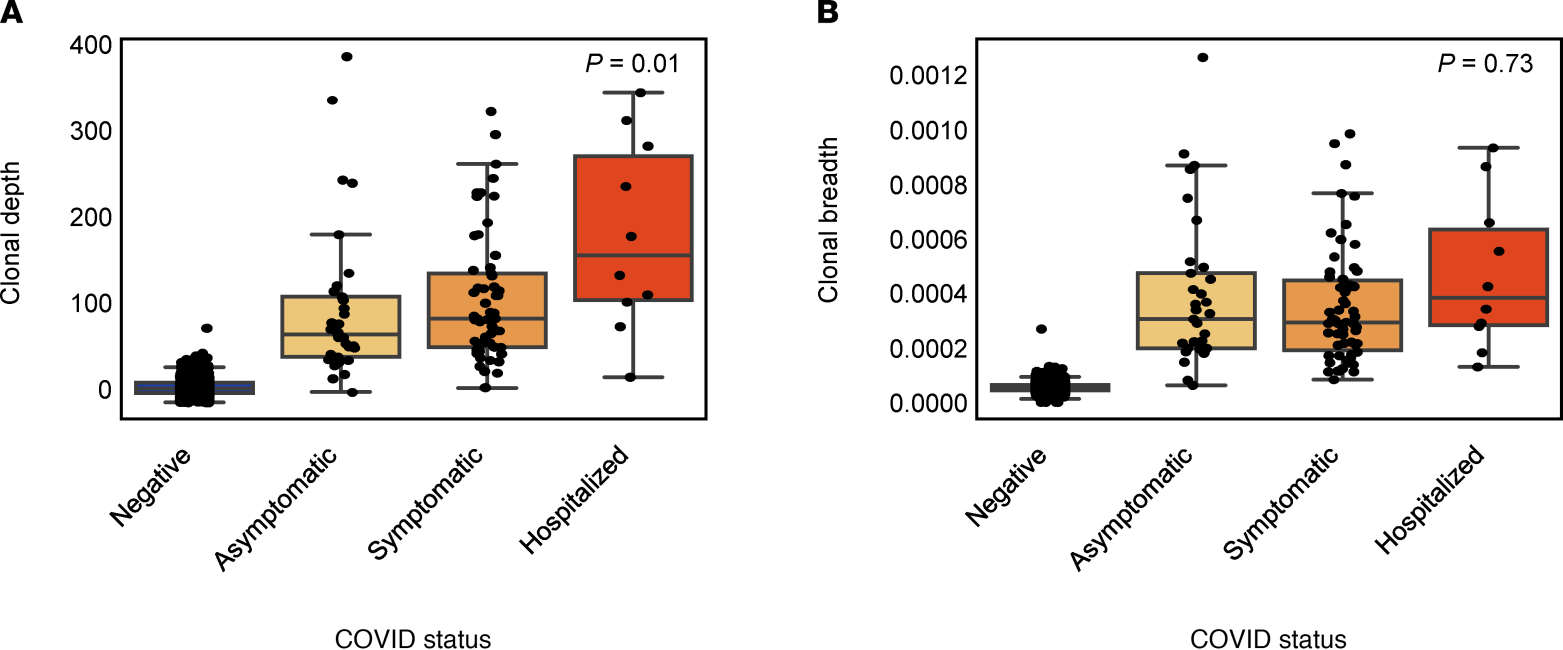
T cell depth and breadth compared across 2022 confident COVID-19^–^ individuals, and in 101 COVID-19^+^ individuals faceted by disease severity. (**A** and **B**) Clonal depth indicates the relative proportion of T cells that are SARS-CoV-2 specific, and clonal breadth indicates the fraction of all unique TCR DNA clones that are SARS-CoV-2 specific. *P* values correspond to Jonckheere’s 2-sided trend test across the 3 PCR^+^ categories. Data are expressed as median ± IQR.

Two of the 2 COVID-19^+^ individuals with a negative T cell test (Figure 1C) result were asymptomatic, indicating that the T cell signal may vary with disease severity. To investigate this possibility, we next assessed the clonal depth and breadth of the T cell response to SARS-CoV-2 in comparison with disease severity. Depth and breadth were calculated as defined previously (18), where breadth measures the relative number of distinct SARS-CoV-2–associated T cell clonotypes, and depth measures the extent to which clonotypic T cells have expanded. Clonal depth was significantly lower among individuals who reported an asymptomatic infection, and it increased in symptomatic and hospitalized individuals (Figure 2A), while clonal breadth did not show strong trends (Figure 2B). This association was present both in older (>60 years) and younger (≤60 years) individuals (Supplemental Figure 2). These results suggest that clonal depth of T cells, reflecting the total number of cells that expanded to defend against the infection, may be a more direct measure of response than the clonal breadth. These results are consistent with other recent findings that the magnitude of T cell response is higher in symptomatic individuals, and these differences may persist for at least 6 months (37).

### Comparisons of the T cell and antibody responses across antigens.

B cell measurements correlate with protective immunity at a population level (38, 39), though, to date, no test has been accepted as a definitive measurement of protective immunity against SARS-CoV-2 for any given individual. This is likely due to other complex factors, including the T cell response that initiates the immune response. However, the extent to which T cell measurements correspond to the B cell response and to protective immunity in SARS-CoV-2 infection is not completely understood. Helper T cells play an important role in initiating the B cell response, while cytotoxic T cells engage independent cellular immune pathways. Additionally, NAbs, which are antibodies that bind to the virus and specifically block infection, are largely directed against the SARS-CoV-2 spike protein (40), while the serology tests compared in this study target either spike (LIAISON SARS-CoV-2 S1/S2 IgG, DiaSorin Molecular) or nucleoprotein (Elecsys Anti-SARS-CoV-2 assay, Roche Diagnostics; ARCHITECT SARS-CoV-2 IgG, Abbott Laboratories), necessitating analysis accounting for these factors. Thus, to investigate this question, we examined NAb titers assayed in 88 individuals who were positive by real-time PCR or by all 4 tests in the original survey (the 3 serology assays and T cell test), enabling comparison across assays without missingness — first comparing the overall T cell signal and then breaking analyses out by cell type and antigen specificity.

**Figure 3:**
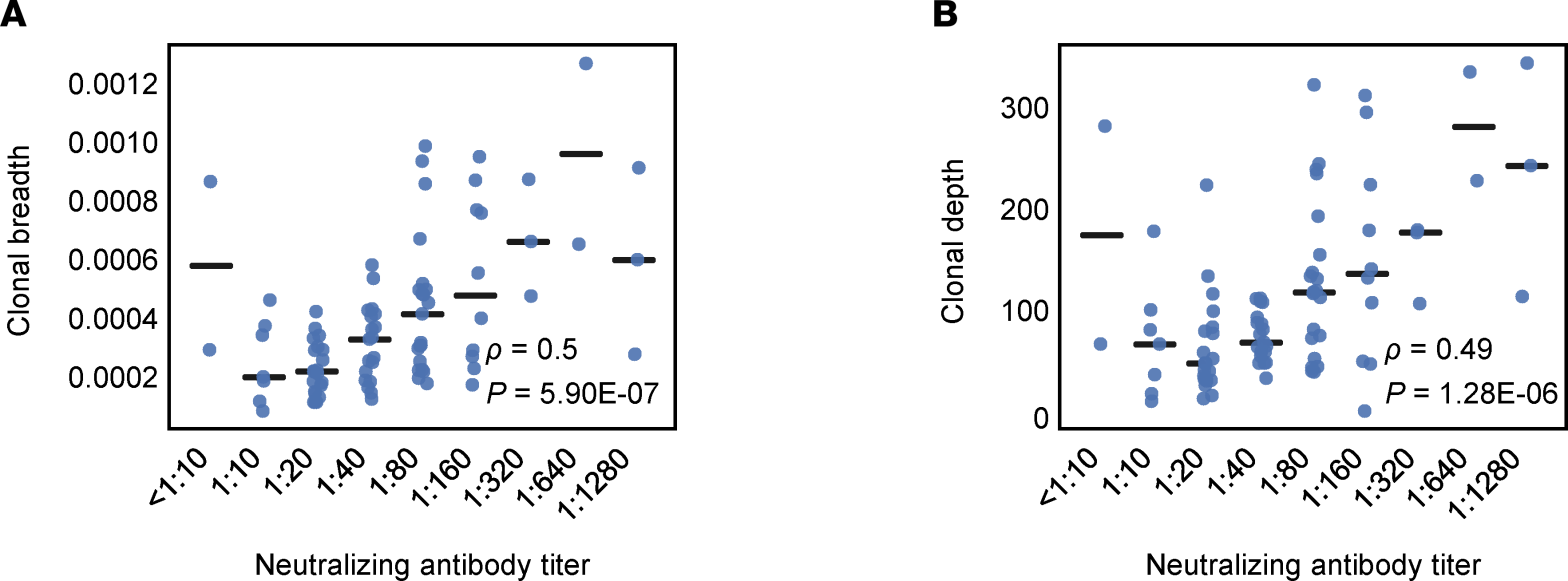
Clonal breadth and depth compared across cases. (**A** and **B**) Clonal breadth and depth compared across 88 cases that were positive by real-time PCR and/or all 4 additional tests and faceted by neutralizing antibody titer. Spearman’s correlations are indicated by *ρ* and corresponding *P* values by *P*.

We found that both clonal breadth and depth of T cells were correlated with NAb titer (Figure 3, A and B). The correlation to NAb titers was comparable with the range from serology testing (higher than the level seen for Roche and lower than that seen for DiaSorin and Abbott; Supplemental Figure 3), despite being only an indirect measure of antibody response. Two very different molecular measurements (T cells versus antibody titers) being correlated suggests that aspects of the overall adaptive immune response can be inferred using just T cells in the case of SARS-CoV-2 infection. Further research to compare T cell signals to clinical outcomes will be needed to establish their utility as a potential correlate of immunity.

To examine different components of the T cell signature in more detail, we next characterized the cell type and antigen specificity of the public TCR sequences that we identified to diagnose SARS-CoV-2 infection (18). For many of the public TCRs and related sequences, direct observation in independent multiplexed antigen-stimulation experiments allowed us to assign TCR sequences to a specific target (41). Of the 4287 public TCR sequences, we identified 1776 and 1605 as CD8^+^ and CD4^+^ sequences, respectively, including 769 CD4^+^-associated sequences to spike protein and 836 CD4^+^-associated sequences to all other proteins.

We next compared and contrasted the breadth and depth of the CD4^+^ and CD8^+^ T cells with the B cell response. We found that the breadth of CD4^+^ T cells with antigen assignments was positively correlated with antibody titers (DiaSorin: Spearman’s *ρ* = 0.5, *P* = 3 × 10^–6^; NAb: Spearman’s *ρ* = 0.6, *P* = 2 × 10^–8^; Abbott: Spearman’s *ρ* = 0.5, *P* = 8 × 10^–6^; Roche: Spearman’s *ρ* = 0.2, *P* = 0.03; Supplemental Figure 4). Similar correlations were observed between antibody titers and the depth of the CD4^+^ T cell response (Supplemental Figure 5). Conversely, there was no correlation between CD8^+^ T cell breadth and antibody levels (DiaSorin: Spearman’s *ρ* = 0.01, *P* = 0.92; NAb: Spearman’s *ρ* = –0.03, *P* = 0.8; Abbott: Spearman’s *ρ* = –0.06, *P* = 0.59; Roche: Spearman’s *ρ* = –0.09, *P* = 0.38). These results underscore the role of helper T cells in supporting the generation of antibodies.

We then explored the potential association of spike-specific signal between T cells and antibodies. DiaSorin IgG spike and NAb titers were more significantly correlated with spike-specific CD4^+^ T cell breadth as compared with non-spike-specific CD4^+^ T cell breadth (Supplemental Figure 6). The breadth of spike-specific CD4^+^ T cells had a partial Spearman’s correlation of 0.5 (*P* = 7 × 10^–6^) and 0.3 (*P* = 4 × 10^–3^) to the DiaSorin IgG spike and NAb titers, respectively. Similar results were observed for clonal depth of spike-specific CD4^+^ T cells (Supplemental Figure 7). On the contrary, when we examined the association with the nucleocapsid phosphoprotein (NP), no significant correlation between spike-specific T cell signal and the Abbott and Roche anti-NP titers was observed. Conversely, the breadth of non-spike-specific CD4^+^ T cells had a partial Spearman correlation of 0.3 (*P* = 0.006) and 0.2 (*P* = 0.04) to the Abbott and Roche anti-NP titers. Overall, these results indicate that robust antibody-mediated immunity is associated with increased diversity of the antigen-specific CD4^+^ T cell repertoire. These results also suggest that the information obtained from the TCR repertoire data may extend beyond the direct measure of the cellular immune response and help simultaneously dissect the concomitant humoral immune response.

### Longitudinal T cell response to SARS-CoV-2.

**Figure 4:**
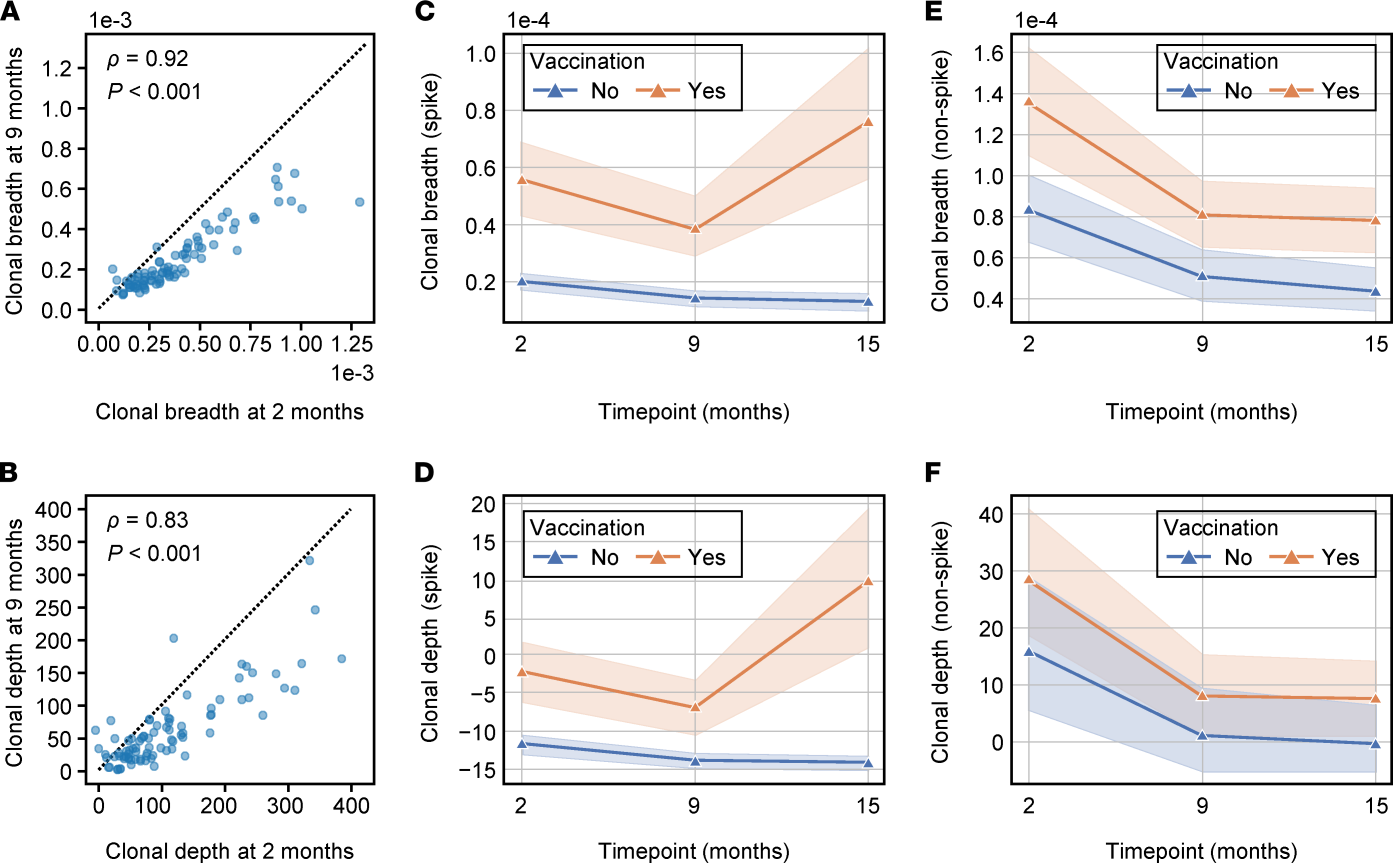
Longitudinal assessment of clonal depth and breadth. (**A** and **B**) Clonal breadth (**A**) and depth (**B**) for COVID cases (*n* = 86) at 2 months and 9 months. The dotted line indicates the line *y* = *x*. Spearman’s correlations are indicated by *ρ* and corresponding *P* values by *P*. (**C**–**F**) Mean clonal breadth and mean clonal depth of T cells specific to spike protein are shown across time, while **E** depicts mean clonal breadth and **F** depicts mean clonal depth of T cells specific to non-spike proteins across time. The shaded areas represent the 95% CI of the mean. Samples were grouped by vaccination status at month 15.

We next sought to characterize the persistence of the T cell response over time in the 101 COVID-19^+^ samples from the initial sample collection. We performed additional T cell sequencing on 86 samples at 9 months and 72 samples collected at 15 months from these same individuals. At 9 months, 90.7% still had a positive T cell test. T cell signal was highly correlated between the 2 time points (Spearman’s *ρ* = 0.83 for clonal depth and Spearman’s *ρ* = 0.92 for clonal breadth), indicating that the magnitude of the initial memory response can be predictive of lasting T cell signal (Figure 4, A and B). In the subset of cases that were asymptomatic, a slightly lower proportion had a positive T cell test at 9 months: 84.6% (22 of 26).

Importantly, vaccines were available in Vo’ by June 2021, when the 15-month samples were collected. Of the 72 samples collected at 15 months, 29 came from individuals who had not received any vaccine dose; 86.2% of these individuals still had a positive T cell test. In the subset of cases that were asymptomatic, the proportion positive by the T cell test decreased to 75% (6 of 8) at 15 months. The high proportion of cases, including asymptomatic ones, with a detectable T cell response at 15 months after infection indicates the strong durability of the memory T cell compartment and persistence of the T cell signal. NAb titers were available longitudinally on a large subset of the cases. Clonal depth and breadth continued to correlate with neutralization at each time point (Supplemental Figure 8).

The vaccinated individuals all had positive T cell tests at 15 months, which provided us the opportunity to investigate the T cell response to vaccination. All of the vaccines administered in the Vo’ participants elicit a response against the SARS-CoV-2 spike protein but not against the viral proteins coded by the rest of the viral genome. In vaccinated individuals, we reasoned that the T cells targeting spike should rebound after the vaccination, while T cells directed against non-spike proteins should continue their slow declining trend. We again took advantage of our prior data (18) to characterize the antigen specificity of T cells in COVID-19cases faceting samples by their vaccination status at 15 months (Figure 4, C–F). Indeed, though still largely detectable, both spike and non-spike T cells waned over time in individuals who had a prior COVID-19infection and were not vaccinated (tests for negative trend: *P* < 0.001 and *P* < 0.001, respectively). In vaccinated COVID-19cases, however, spike T cells significantly increased both in breadth and depth after vaccination (comparison of month 9 to month 15: *P* < 0.001 and *P* < 0.001, respectively), whereas non-spike T cell clonal depth and breadth continued to decline (test for trend: *P* < 0.001). We did observe significant differences in the clonal depth and breadth of spike T cells between vaccinated and unvaccinated cases, even at prevaccine time points (all *P* < 0.02), likely due to biases in age, disease severity, and possibly other factors in the subset of individuals who elected early vaccination (Supplemental Table 2). However it is unlikely that these differences could explain the significant increases in clonal depth and breadth observed after vaccination. A similar trend was observed in antibody levels (24), and this further supports the vaccine-related changes observed in T cells.

## Discussion

In this study, we present evidence — gathered in an unbiased and carefully annotated population — that the T cell response, as assessed by immunosequencing, is a highly sensitive and specific indicator of prior SARS-CoV-2 infection. We found that T cell responses were associated with clinical severity and with NAb levels and that they were boosted by vaccination. Importantly, they were present at least 2 months after infection, even in cases that were asymptomatic, and they persisted 15 months after the initial infection in a majority of individuals.

The findings that T cell immunity has similar sensitivity to the antibody response in detecting SARS-CoV-2 infection have important conceptual and diagnostic implications; however, they are not entirely unexpected. Previous experiences with the related MERS and SARS-CoV-1 infections demonstrated that coronavirus-specific T cells have long-term persistence and contribute to protection even in individuals without seroconversion (28–30). Recent evidence suggests that a similar pattern is present during SARS-CoV-2 infection (14, 42, 43).

The structure of the T cell repertoire, in terms of diversity and clonal expansion, also provides important information to promote understanding some of the differences that characterize symptomatic and asymptomatic individuals. Our findings demonstrate that the T cell response correlated with prior disease symptoms and severity at a convalescent time point over 2 months after infection, suggesting — as potential hypotheses — that differences in viral load or viral persistence during acute infection correlate both with symptoms and the depth of the T cell response. This elevated signal persisted over time and was detected at 15 months, though at slightly lower levels. In addition, information gathered from the TCR repertoire analysis also correlates with the humoral responses to the virus, providing insight on the overall immune system response and possible defense against SARS-CoV-2. The T cell response from helper T cells strongly correlated with NAb titer, a potential measure of protection. Helper T cells recognizing spike protein antigens correlated more strongly with the overall antibody levels to the same protein but not other proteins, suggesting that antigen-specific CD4^+^ T cell help may be required for robust development of antibodies during viral infections (44). These correlations between helper T cells and NAbs may provide evidence relevant to disease pathology, as it has not been established whether the T cell response is exclusively beneficial or whether it might also contribute to immunopathology (11).

Our findings also have important implications for measuring response to vaccines, as well as to natural infection, since the broad set of TCRs measured in this assay allows for separating responses to spike, which is the primary target of most current vaccines, from other viral proteins. Indeed, the T cell response was sensitive to changes elicited by the current generation vaccines targeting the SARS-CoV-2 spike protein, in line with previous observations using T cell functional assays (45). Clonal breadth and depth for sequences inferred to target the spike protein significantly increased after vaccination at the 15-month time point in cases receiving 1 or more vaccines, but they did not increase for sequences outside the spike. Additionally, no enhancement in depth and breadth was evident in individuals who were unvaccinated.

Correlations of T cell responses with disease severity, antibody measurements, and vaccination are promising, but additional validation studies with orthogonal T cell assays are needed to confirm the extent to which the depth and breadth of the public T cell response, as measured by immunosequencing, capture the overall T cell response. T cell phenotype and function represent another important dimension that has been shown to impact disease severity (46). Since bulk immunosequencing does not capture functional information, this limitation will need to be addressed in future studies. Finally, additional studies on novel SARS-CoV-2 variants are needed to assess how robust the current set of SARS-CoV-2–associated TCRs are to changes in SARS-CoV-2 as the virus evolves over time, though an early study examining the overlap of novel variants and previously generated T cell/epitope maps is promising (47).

Given the continued emergence of new viral variants capable of at least partial evasion of vaccine-induced immunity (23), there is a strong need for correlates of protection against severe disease. While antibody titers correlate with disease severity and with the probability of breakthrough infection after vaccination, they are not sufficiently predictive to inform individuals of their COVID-19 risk (38, 39). Despite the limitations discussed above, our data suggest that T cells represent another key component of the adaptive immune response that correlates with important clinical factors, including disease severity. Mounting evidence also suggests that, while antibody-derived immunity is severely impacted by SARS-CoV-2 evolution (23, 24, 48), the T cell response may be more robust against viral variants (25, 47, 49). Though tools exist to study T cells in a research setting, they remain difficult to quantify accurately at population scale (50). T cell immunosequencing, which requires only genomic DNA as an input, is thus an attractive and scalable tool for use in clinical applications.

## Methods

### Clinical cohort and sample collection.

This report extends results for the Vo’, Italy, cohort initially described in Lavezzo et al. (35). Upon the detection of SARS-CoV-2 in a deceased resident of Vo’ on February 21, 2020, an epidemiological study was conducted to investigate the prevalence of SARS-CoV-2 infection in the municipality. Sampling for viral PCR testing was performed on the majority of the population immediately after the detection of the first cases (February 21–29, 2020) and again at the end of a 2-week lockdown (March 7, 2020). Follow-up serum and whole blood samples were collected 56 days later (month 2 time point) in early May, at month 9, and again at month 15 for antibody serology and T cell testing. Antibody response was measured using 3 commercial serology testing kits: LIAISON SARS-CoV-2 S1/S2 IgG (DiaSorin Molecular), Elecsys Anti-SARS-CoV-2 assay (Roche Diagnostics), and ARCHITECT SARS-CoV-2 IgG (Abbott Laboratories). Manufacturer cutoffs were used for determining if samples were positive by each test. Serum samples were also used to perform microneutralization assays; more detail on the antibody testing methods is in Dorigatti et al. (36).

In addition to biospecimen collection, clinical data were collected for each study participant, including the results of SARS-CoV-2 testing, demographics, health records, and residence and contact network information. The definition of symptomatic used in this study is a participant who required hospitalization and/or reported fever (yes/no or a temperature above 37°C), and/or cough, and/or at least 2 of the following symptoms: sore throat, headache, diarrhea, vomit, asthenia, muscle pain, joint pain, loss of taste or smell, or shortness of breath. Symptomatic cases that reported hospitalization are split out separately as “hospitalized” in the disease severity analyses.

### Immunosequencing of TCR repertoires.

Genomic DNA was extracted from frozen, plasma-depleted blood samples using the Qiagen DNeasy Blood Extraction Kit (Qiagen). As much as 18 μg of input DNA was then used to perform immunosequencing of the third complementarity determining (CDR3) regions of TCR-β chains using the ImmunoSEQ Assay (Adaptive Biotechnologies). Briefly, input DNA was amplified in a bias-controlled multiplex PCR, followed by high-throughput sequencing. Sequences were collapsed and filtered to identify and quantitate the absolute abundance of each unique TCR-β CDR3 region for further analysis, as previously described (32, 51, 52). In order to quantify the proportion of T cells out of total nucleated cells input for sequencing, or T cell fraction, a panel of reference genes present in all nucleated cells was amplified simultaneously (53).

### Characterization of the T cell response.

Classification of prior infection with SARS-CoV-2, as well as the clonal depth and breadth of T cell response, were calculated using a method similar to prior work (18). Briefly, TCR repertoires from 784 unique cases of real-time PCR–confirmed SARS-CoV-2 infection and 2447 healthy controls collected before 2020 were compared by 1-tailed Fisher’s exact tests to identify 4469 public TCR-β sequences (enhanced sequences) significantly enriched in SARS-CoV-2^+^ samples (all training data to identify the enhanced sequences for SARS-CoV-2 infection came from multiple other study cohorts and not the population being analyzed here). The enhanced sequences were used to develop a classifier predicting current or past infection with SARS-CoV-2 using a simple 2-feature logistic regression with independent variables E and N, where E is the number of unique TCR-β DNA sequences that encode an enhanced sequence and N is the total number of unique TCR-β DNA sequences in that subject. Application of this initial clinical classifier to this study demonstrated the high sensitivity (97%) reported above.

We have since developed a method to improve specificity near the decision boundary of the logistic regression by filtering enhanced sequences that may be potential false positives. Specifically, TCRs that are likely associated with CMV or with multiple antigens in different HLA backgrounds — and, thus, not truly diagnostic of SARS-CoV-2 infection — are identified by Fisher’s exact testing on TCR-β repertoires of ~2000 healthy controls with available HLA genotyping and CMV serotyping data. From this list of ~1.8 million sequences, the 182 sequences that were also identified as SARS-CoV-2–enhanced sequences were removed, leaving 4287 enhanced sequences. The 2-feature logistic regression classifier was refitted to the original training data using this pruned enhanced sequence list, and a decision boundary representing 99.8% specificity on 1657 controls was used to define the test-positive threshold used in the present study. The pruned list of enhanced sequences was also used to calculate the clonal depth and breadth using the same formulae as in Snyder et al. (18).

### Antigen-specific assignment of TCRs.

We assigned public TCRs to antigens and CD4^+^ or CD8^+^ cellular phenotypes by cross-referencing enhanced sequences identified via our case/control design with TCRs observed in multiplexed antigen-stimulation experiments, both described in prior work (18). Briefly, in these experiments, viral peptide panels were designed separately for class I and class II HLA binding. Peptides in each panel were pooled in a combinatorial fashion as described previously (41). T cells from COVID-19^+^ donor PBMCs were expanded, stimulated with the peptide panel pools, and then sorted into activated CD4^+^ or CD8^+^ subsets for sequencing.

To maximize the number of TCR antigen assignments, we identified a set of public TCRs from an augmented sample of repertoire data. We combined the training and validation repertoires with an additional 1143 COVID^+^ samples accrued since this model was developed and included samples from this study that were identified as COVID-19^–^ by our T cell test as controls. Our final sample of repertoires consisted of 1927 cases and 4135 controls. We identified ~500,000 public TCRs with a Fisher’s Exact Test (FET) *P* <0.05. At this level of significance, we expected a significant fraction of the public TCRs to be false positives; however, we cross-referenced this list of TCRs with a set of ~400,000 TCRs that was independently derived from our antigen-stimulation experiments, yielding 3381 overlapping TCRs. The fraction of false-positive TCRs in this overlapping set was significantly smaller. These 3381 overlapping TCRs had antigen protein and CD4^+^/CD8^+^ assignments determined from our antigen-stimulation experiments.

### Data and materials availability.

Clinical data and T cell repertoire profiles are available as part of the ImmuneCODE data resource (54) and can be downloaded from the Adaptive Biotechnologies immuneACCESS site under the immuneACCESS Terms of Use at https://clients.adaptivebiotech.com/pub/gittelman-2022-jci

### Statistics.

Statistical methods used for identification of SARS-CoV-2–associated TCRs and for assignment of public TCRs to antigens are described above. We used Jonckheere’s 2-sided trend test to evaluate ordered differences in clonal depth or breadth among COVID-19^+^ individuals grouped by disease severity. *P* values less than 0.05 were considered significant.

We calculated the Spearman’s rank correlations between antibody titers and CD4^+^ and CD8^+^ T cell response using the Pingouin package in Python (55) and reported the 2-sided significance. We noted that the spike-specific CD4^+^ T cell signal correlates with the non-spike-specific signal (Spearman’s *ρ* = 0.5, *P* = 2 × 10^–6^; Supplemental Figure 5). To disentangle the confounding correlations, we calculated partial Spearman’s rank correlations between spike and non-spike specific T cell response and antibody titers and reported the 2-sided significance. The partial correlation coefficients and *P* values are denoted with tildes, appearing above the significance symbol, when reporting them in the figures. We examined the CD4^+^ T cell response specific to spike and all other assayed proteins. When calculating the partial correlation between the antibody titers of 1 test and spike-specific T cell response, we took the non-spike protein–specific responses as a covariate, and vice versa. The partial correlations we calculated characterize the correlation between 2 variables that cannot be explained by the covariates and, thus, is conservative.

For longitudinal analyses of vaccinated or unvaccinated individuals, we used generalized estimating equation models with exchangeable covariance structure to account for correlation over time and adjusted for age, sex, and COVID-19severity. To assess differences between the 9- and 15-month time points, we used linear regression models adjusted for age, sex, and COVID-19severity and clustering on study participant in vaccinated or in unvaccinated individuals.

### Study approval.

The first, second, and third surveys of the Vo’ population were approved by the Ethics Committee for Clinical Research of the province of Padova. Study participation was approved by written informed consent. For participants younger than 18 years, consent was provided by a parent or legal guardian.

## Author contributions

Order of co–first authors was assigned alphabetically. TMS, EL, IK, LB, DMC, PG, ST, JMC, HSR, GT, and AC conceptualized the study. Data curation was performed by EL, LM, and ST (clinical and serology); by IK and HSR (T cell repertoire); and by EF, CDV, M Pacenti, CB, MC, F Saluzzo, AP, M Plebani, F Simeoni, JB, NIL, and DL (laboratory testing). RMG, TMS, HJZ, RE, and CLC conducted data analyses, and SD and LB contributed to clinical interpretation. TMS, EL, GT, HSR, and AC supervised the study. Data visualizations were created by RMG, HJZ, and RE. TMS, HMC, HSR, GT, and RMG wrote the original draft of the manuscript, and RMG, SD, IK, EL, ST, DMC, PG, AC, and CLC reviewed and edited the manuscript.

## Supplementary Material

Supplemental data

ICMJE disclosure forms

## Version 1 04/19/2022

In-Press Preview

## Version 2 05/23/2022

Electronic publication
