# Supplementary material for: Longitudinal analysis of T cell receptor repertoires reveals shared patterns of antigen-specific response to SARS-CoV-2 infection
Source: JCI Insight. 2022 May 23;7(10):e151849. doi: 10.1172/jci.insight.151849 (PMC9220833; doi:10.1172/jci.insight.151849)
Supplement: Supplemental data [file jciinsight-7-151849-s199.pdf]

Longitudinal analysis of T-cell receptor repertoires reveals shared patterns of antigen-specific response to SARS-CoV-2 infection

Rachel M. Gittelman[1@], Enrico Lavezzo[2@], Thomas M. Snyder[1@], H. Jabran Zahid[3], Cara L. Carty[1], Rebecca Elyanow[1], Sudeb Dalai[1,4*], Ilan Kirsch[1], Lance Baldo[1], Laura Manuto[2], Elisa Franchin[2], Claudia Del Vecchio[2], Monia Pacenti[5], Caterina Boldrin[5], Margherita Cattai[5], Francesca Saluzzo[6], Andrea Padoan[7], Mario Plebani[7], Fabio Simeoni[8], Jessica Bordini[9], Nicola I. Lorè[6], Dejan Lazarevic[8], Daniela M. Cirillo[6], Paolo Ghia[9,10], Stefano Toppo[2,11], Jonathan M. Carlson[3], Harlan S. Robins[1**], Andrea Crisanti[2,12**], Giovanni Tonon[8,9,10**]

[1]Adaptive Biotechnologies, Seattle, WA, USA.

[2]Department of Molecular Medicine, University of Padova, Padua, Italy.

[3]Microsoft Research, Redmond, WA, USA.

[4]Division of Infectious Diseases and Geographic Medicine, Stanford University School of Medicine, Stanford, CA, USA.

[5]Azienda Ospedale Padova, Microbiology and Virology Unit, Padua, Italy.

[6]Division of Immunology, Transplantation and Infectious Disease, IRCCS Ospedale San Raffaele, Milan, Italy.

[7]Department of Medicine, University of Padova, Padua, Italy.

[8]Center for Omics Sciences, IRCCS Ospedale San Raffaele, Milan, Italy.

[9]Division of Experimental Oncology, IRCCS Ospedale San Raffaele, Milan, Italy.

[10]Università Vita-Salute San Raffaele, Milan, Italy.

[11]CRIBI Biotech Center, University of Padova, Padua, Italy.

[12]Department of Life Sciences, Imperial College London, London, UK.

[@]These authors share the first author position and are listed in alphabetical order.

*Author was an Adaptive employee at time of research.

Supplemental Materials

Figure S1. Evidence of SARS-CoV-2 infection in ground truth individuals without a supporting PCR test

Figure S2. T-cell signatures stratified by disease severity and age.

Figure S3. Quantitative serology scores for DiaSorin (A), Abbott (B) and Roche (C) are compared across 88 individuals and faceted by Neutralizing Antibody Titer.

Figure S4. Correlation between CD4+ T-cell breadth and four antibody tests.

Figure S5. Correlation between CD4+ T-cell depth and four antibody tests.

Figure S6. Correlation between spike-specific and non-spike CD4+ T-cell breadth and antibody signals.

Figure S7. Correlation between spike-specific and non-spike CD4+ T-cell depth and antibody signals.

Figure S8. Mean clonal depth and breadth of Sars-CoV-2 spike and non-spike sequences over time, stratified by vaccination status at 9 months and 15 months

Table S1. Comparison of T-cell and serology test results in 3 subject groups.

Table S2. Demographics of individuals by vaccination status at 15 months.

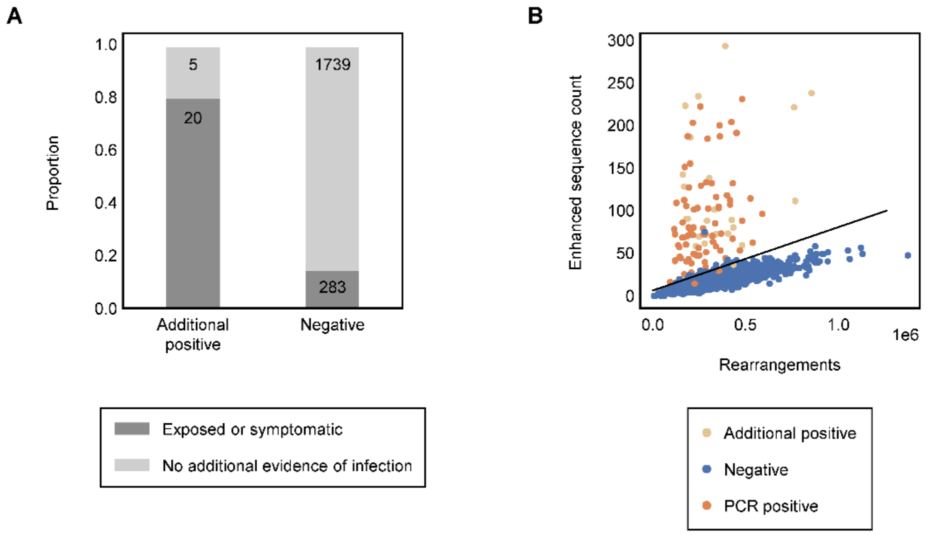

Supplemental Figure S1. Evidence of SARS-CoV-2 infection in ground truth individuals without a supporting PCR test. (**A**) The rate of individuals that were exposed (resided in the same household as a positive case) or symptomatic in the group of additional samples that were positive by the ground truth case definition in Dorigatti et al (51) despite a lack of PCR evidence, compared to individuals that were confidently COVID-19 negative by PCR and all 3 serology tests. Numbers indicate the raw sample numbers in each category. (**B**) Comparison of the T-cell signal in individuals that were positive by PCR (dark orange), positive by the additional ground truth definition (light orange), or confidently negative by PCR and all 3 serology assays (blue).

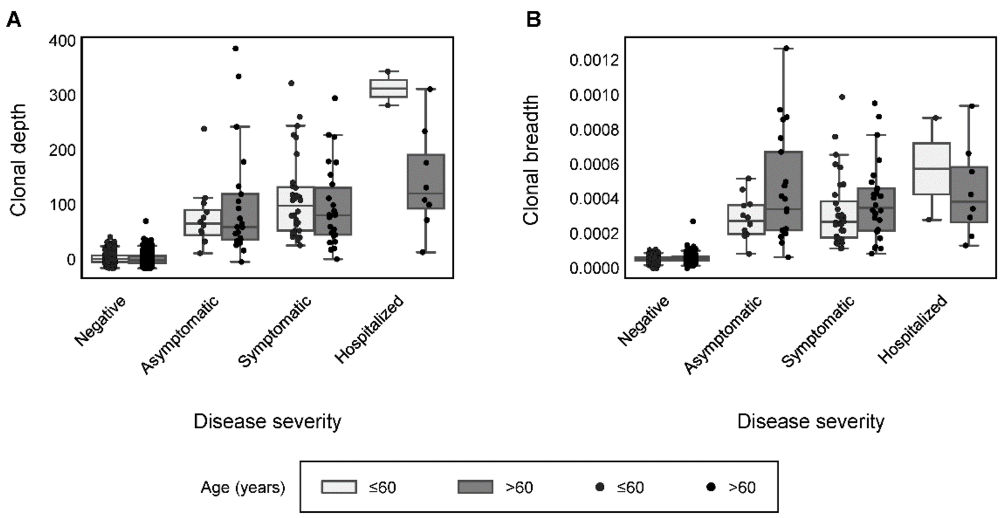

Supplemental Figure S2. T-cell signatures stratified by disease severity and age. PCR- samples without additional evidence of SARS-CoV-2 infection are plotted alongside PCR+ samples stratified by disease severity, and age categorized as ≤60 years and >60 years. Data are expressed as median ± interquartile ranges.

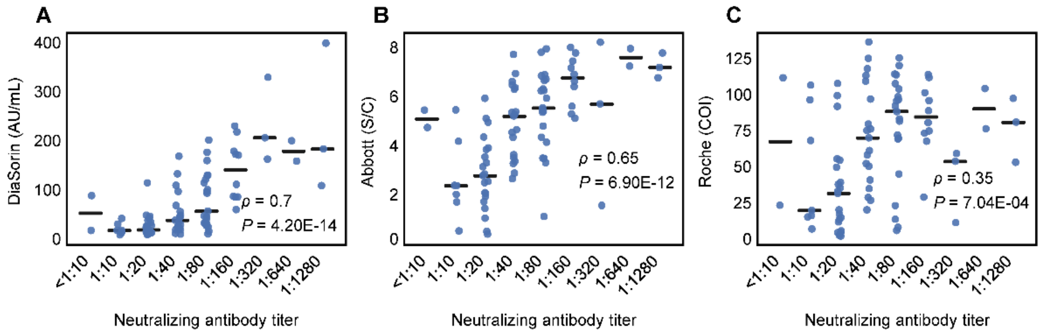

Supplemental Figure S3. Quantitative serology scores for DiaSorin (A), Abbott (B) and Roche (C) are compared across 88 individuals and faceted by neutralizing antibody titer. Spearman correlations are indicated by ***ρ*** and corresponding *P* values by *P*. Horizontal lines indicate means. COI, cutoff index; S/C, signal/cutoff index.

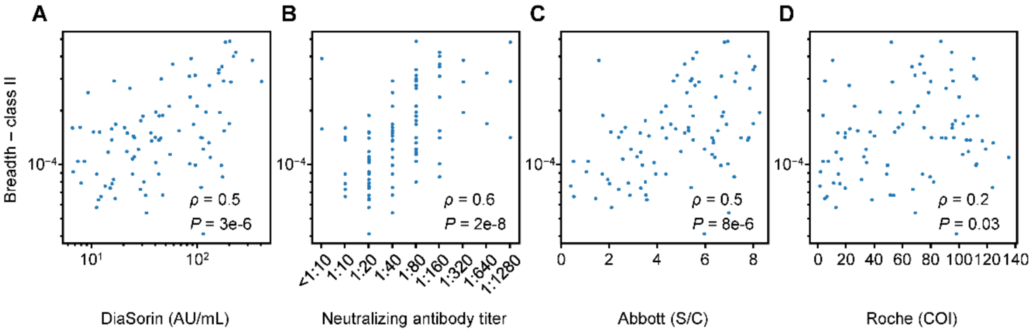

Supplemental Figure S4. Correlation between CD4+ T-cell breadth and four antibody tests. Spearman correlations are indicated by $\boldsymbol{\rho}$ and corresponding *P* values by *P*. COI, cutoff index; S/C, signal/cutoff index.

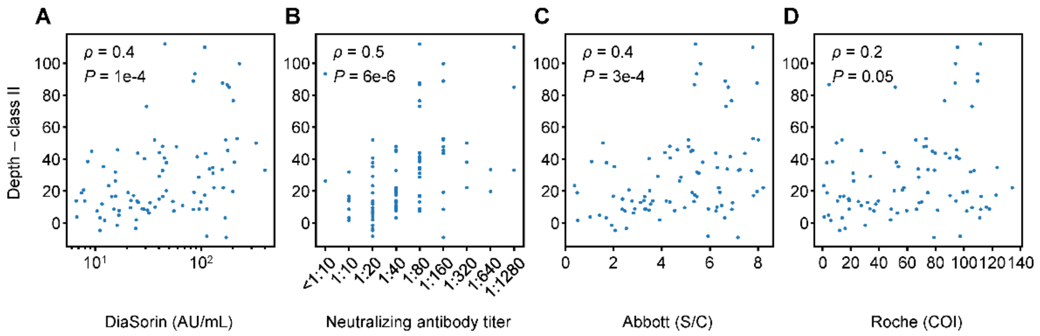

Supplemental Figure S5. Correlation between CD4+ T-cell depth and 4 antibody tests.

Spearman correlations are indicated by ***ρ*** and corresponding *P* values by *P*. COI, cutoff index; S/C, signal/cutoff index.

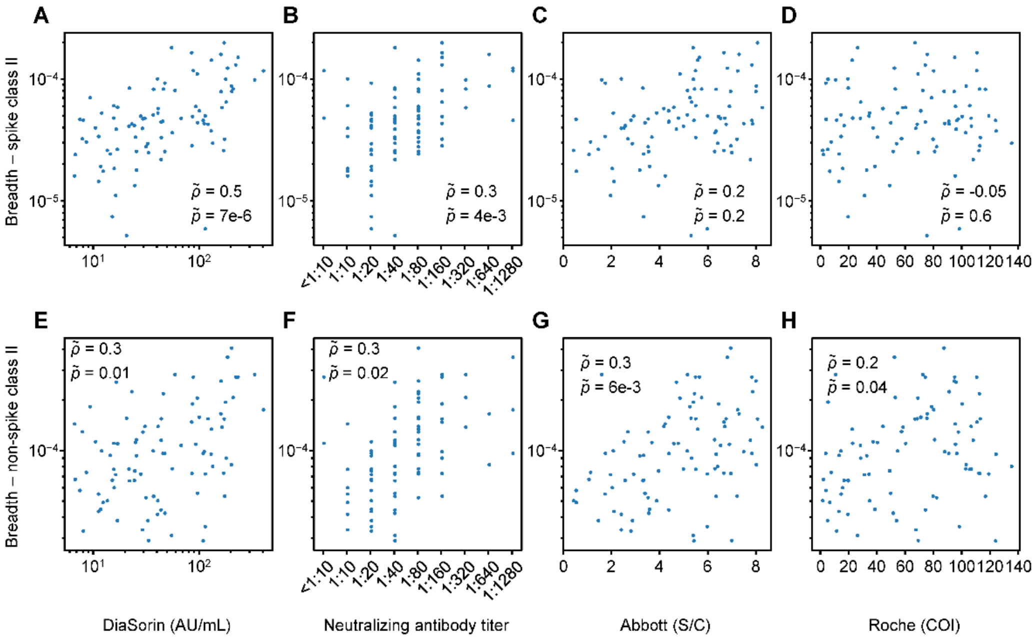

Supplemental Figure S6. Correlation between spike-specific (A-D) and non-spike CD4+ T-cell breadth and antibody signals (E-F). Partial correlation analysis was performed across 2 T-cell sequence sets (Class II spike sequences and all other identified antigen-specific sequences) as well as 4 antibody tests: DiaSorin (**A**, **E**) and neutralizing antibody titer (**B**, **F**) for spike protein, and Abbott (**C**, **G**) and Roche (**D**, **H**) for nucleocapsid phosphoprotein. COI, cutoff index; S/C, signal/cutoff index.

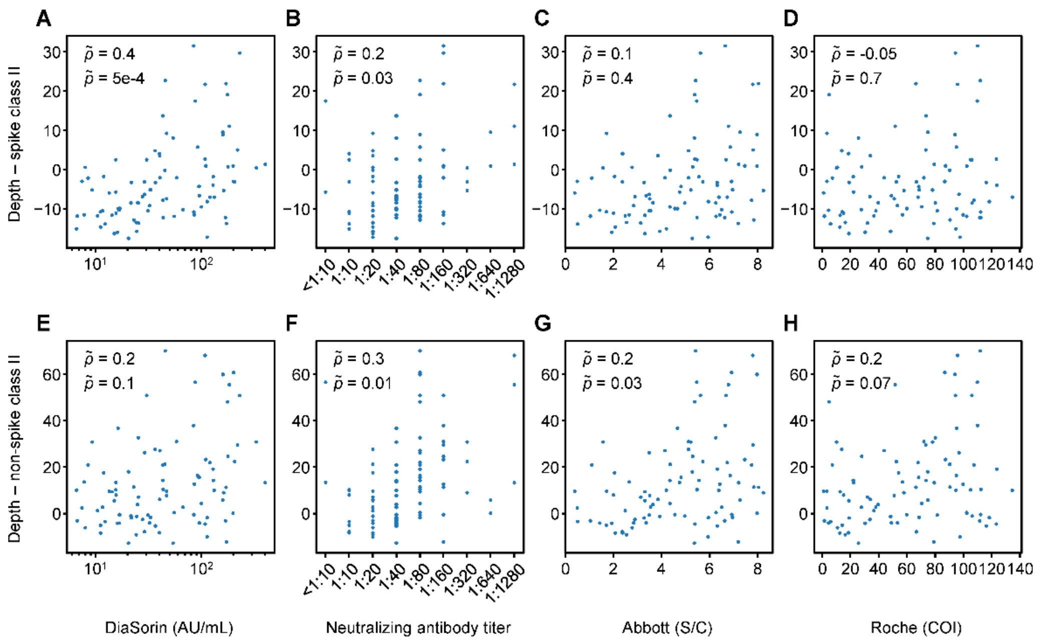

Supplemental Figure S7. Correlation between spike-specific (A-D) and non-spike CD4+ T-cell depth and antibody signals (E-F). Partial correlation analysis was performed across 2 T-cell sequence sets (Class II spike sequences and all other identified antigen-specific sequences) as well as 4 antibody tests: DiaSorin (**A**, **E**) and neutralizing antibody titer (**B**, **F**) for spike protein, and Abbott (**C**, **G**) and Roche (**D**, **H**) for nucleocapsid phosphoprotein. COI, cutoff index; S/C, signal/cutoff index.

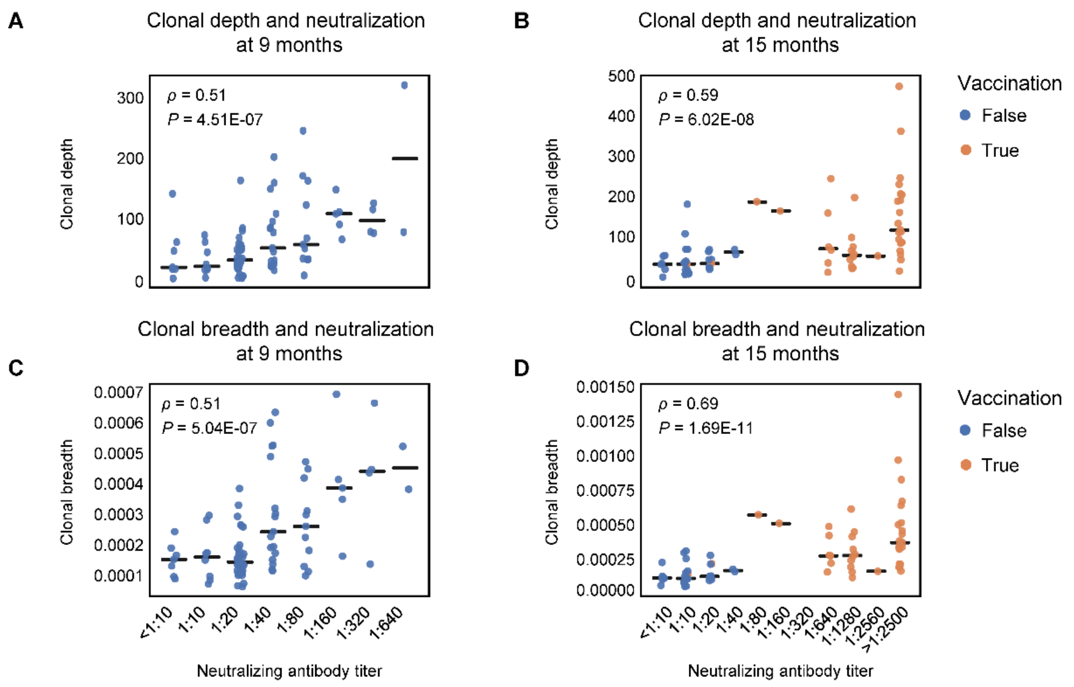

Supplemental Figure S8. Mean clonal depth (A, B) and breadth (C, D) of SARS-CoV-2 spike and non-spike sequences over time, stratified by vaccination status at 9 months (A, C) and 15 months (B, D). Spearman correlations are indicated by ***ρ*** and corresponding *P* values by *P*. Horizontal lines indicate means.

Supplemental Table S1. Comparison of T-cell and serology test results in three subject groups: confirmed PCR+, PCR- who share a household with a PCR+ individual and/or reported symptoms, and PCR- without household exposure or symptoms. Data represent only the samples with data from all antibody assays and T-Detect; 3 DiaSorin calls in the PCR+ group were equivocal and included in sensitivity estimate as false negatives. 95% confidence intervals are calculated via bootstrapping.

	PCR+			PCR- (exposed or symptomatic)			PCR- (no exposure or symptoms)		
	Neg	Pos	Sensitivity (95% CI)	Neg	Pos	% Pos (85% CI)	Neg	Pos	% Neg (95% CI)
T-Detect	1	69	98.6% (95.7–100.0)	258	17	6.2% (3.6–9.5)	1793	18	99.0% (98.5–99.5)
Diasorin (spike)	12	55	78.6% (68.6–87.1)	255	19	6.9% (4.0–9.8)	1777	33	98.1% (97.5–98.7)
Abbott (NP)	5	65	92.9% (87.1–98.6)	261	14	5.1% (2.6–7.6)	1802	9	99.5% (99.1–99.8)
Roche (NP)	0	70	100.0% (100.0–100.0)	262	13	4.7% (2.6–7.3)	1805	6	99.7% (99.4–99.9)

Supplemental Table S2. Characteristics of COVID cases[A] at 2 months, stratified by future vaccination status at Month 15.

Characteristic	Unvaccinated, n = 29	Vaccinated, n = 43	[B]Unknown, n = 29
[C]Age in years, %			
≤10	3.2	0	10.3
11–40	44.8	0	27.6
41–50	17.2	2.3	20.7
51–60	27.6	27.9	17.2
61–70	6.9	34.9	3.4
71–80	3.4	30.2	20.7
>80	0	4.7	0
[C]Female sex, %	72.4	41.9	27.6
[C]COVID severity, %			
Asymptomatic	27.6	37.2	31.0
Symptomatic	72.4	46.5	58.6
Hospitalized	0	16.3	10.3

[A]Based on PCR+ test or positivity to at least 2 SARS-CoV-2 antigens (nucleocapsid protein and spike protein) based on 3 different serology tests at 2 months.

[B]Participant was present at 2-month visit, but not at 15-month visit, so vaccination status is unknown.

[C]Significantly different between unvaccinated and vaccinated cases, $P < 0.05$
